# Error Correction in Bluetooth Low Energy via Neural Network with Reject Option

**DOI:** 10.3390/s25196191

**Published:** 2025-10-06

**Authors:** Wellington D. Almeida, Felipe P. Marinho, André L. F. de Almeida, Ajalmar R. Rocha Neto

**Affiliations:** 1 Department of Teleinformatics Engineering, Technology Center, Federal University of Ceará, Fortaleza 60455-970, Ceará, Brazil; wellington.almeida@alu.ufc.br (W.D.A.); fpmarinho@alu.ufc.br (F.P.M.); andre@gtel.ufc.br (A.L.F.d.A.); 2 Federal Institute of Education, Science and Technology of Ceará, Fortaleza, Federal University of Ceara, Fortaleza 60040-215, Ceará, Brazil

**Keywords:** wireless communication, error correction, neural network, reject option, image error correction

## Abstract

This paper presents an approach to error correction in wireless communication systems, with a focus on the Bluetooth Low Energy standard. Our method uses the redundancy provided by the cyclic redundancy check and leaves the transmitter unchanged. The approach has two components: an error-detection algorithm that validates data packets and a neural network with reject option that classifies signals received from the channel and identifies bit errors for later correction. This design localizes and corrects errors and reduces transmission failures. Extensive simulations were conducted, and the results demonstrated promising performance. The method achieved correction rates of 94–98% for single-bit errors and 54–68% for double-bit errors, which reduced the need for packet retransmissions and lowered the risk of data loss. When applied to images, the approach enhanced visual quality compared with baseline methods. In particular, we observed improvements in visual quality for signal-to-noise ratios between 9 and 11 dB. In many cases, these enhancements were sufficient to restore the integrity of corrupted images.

## 1. Introduction

Efficient data communication remains a significant challenge in wireless systems, particularly in scenarios affected by multipath fading, which contributes to intersymbol interference. These factors degrade the quality of received information in Bluetooth Low Energy (BLE) devices. Traditional approaches to mitigate this problem include equalization and error correction. Although these are distinct techniques, they are closely related and can be used together to reduce the effects of wireless channel propagation [1,2]. Channel equalization is a fundamental method employed to combat intersymbol interference. Concurrently, error correction codes are applied during encoding and evaluated during decoding to correct certain error cases that may arise in received data. While equalizers are generally effective, their performance may become inadequate when the channel experiences rapid variations, since some equalizers require frequent adjustment of their coefficients to track channel dynamics accurately [3].

Neural network algorithms can filter the received signal and classify the patterns that emerge after propagation through the channel, even in scenarios with significant channel variations. The extreme learning machine (ELM) is a neural network algorithm that achieves high accuracy and minimizes errors, even with fixed parameters in the hidden layer [4]. ELM is particularly suitable for systems that require fast training and strong generalization capabilities [5].

Error-correcting codes (ECCs) have been proposed as an alternative for packet recovery at the receiver side. However, ECCs introduce overhead due to redundant bits, which increase the packet size, transmission time, and channel utilization [6]. Regarding memory requirements, for example, Turbo and Low-Density Parity-Check (LDPC) codes demand substantial resources to store large matrices. To achieve improved bit error rate (BER) performance, LDPC codes often require block lengths on the order of thousands of bits [7,8]. The matrix operations for such large codewords demand considerable memory, higher computational resources, and more complex decoding processes [6]. In contrast, cyclic redundancy check (CRC) codes require minimal memory, as they involve only the storage of a checksum calculated from the data [9]. Devices with limited processing power or energy resources, such as IoT sensors, may be unable to handle the computational overhead imposed by ECC. Moreover, conventional ECCs are not supported in BLE due to energy constraints at the transmitter [10]. Instead, CRC codes are widely applied for packet error detection. Unlike ECC, CRC is primarily used for error detection [11]. In this work, we use CRC solely to detect errors in BLE data packets.

A binary communication system is characterized by bit outputs equal to {0, 1}. Errors occur when a bit transmitted as 0 is erroneously received as 1 (or vice versa). Bit errors typically occur when a classifier or equalizer is uncertain about the value represented by the signal. Classifiers with a reject option, however, can assign a rejection (R) label in addition to zero or one in binary classification tasks. This approach rejects patterns that are likely to be misclassified, thereby improving the overall accuracy [12]. In our approach, we combine the binary output of a neural network with a reject option to create a three-output process: 0, 1, or rejection (*R*). The reject option was first proposed by [13], who demonstrated the design of an optimal classifier that balances the rejection and accuracy rates. The reject option has since been applied in various fields, including medical diagnosis [14,15,16], mobile robotics [17], and software defect prediction [18].

**Figure 1:**
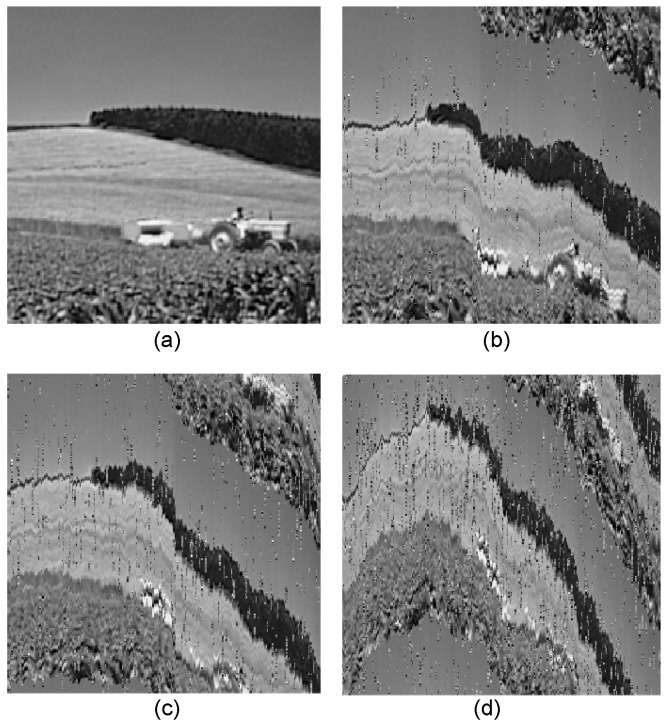
Impact of increasing BER on the JPEG image ‘cornfield’ in BLE transmission: (**a**) original image, (**b**) BER = 0.1%, (**c**) BER = 0.2%, (**d**) BER = 0.4%.

In the context of transmitting compressed images over unreliable channels, quality degradation is a common consequence. When multimedia data are transmitted through error-prone channels or under bandwidth constraints, packet loss due to errors can significantly compromise the quality of the received data [19]. Transmission errors may occur either as isolated bit flips or as bursts of consecutive erroneous bits within a packet. In entropy-coded images, even a single transmission error can propagate and affect subsequent bits [20], as illustrated in Figure 1. Several methods have been proposed for image error concealment; however, the recovery quality depends on the reconstructed region, which is often interpolated from neighboring spatial or temporal content [21]. In BLE wireless environments, retransmission of corrupted packets is generally discouraged due to latency and network congestion constraints.

In recent years, several studies have proposed methods for error correction in applications using Bluetooth Low Energy [10,21,22,23,24,25,26]. A common feature across these works is the reliance on CRC mechanisms, with approaches generally divided into two categories.

Lookup table approaches: Error correction using CRC syndromes has been investigated in [27,28]. These techniques employ precomputed lookup tables generated prior to communication, where each entry corresponds to the syndrome produced by one or more errors at specific positions. The underlying concept is that an error at any packet position yields a unique remainder when the error polynomial is divided by the generator polynomial. The lookup table maps each possible remainder to its corresponding error position. Thus, when a packet with bit errors is received, the observed remainder can be matched against the table to identify the exact error position. This method has been shown to correct single-bit errors [27] and double-bit errors [28] and can in principle be extended to multiple errors by storing all possible error patterns. However, if the packet length exceeds the generator polynomial period, different error positions may produce the same syndrome, leading to ambiguity. Furthermore, these strategies face practical challenges due to their inflexibility and excessive memory requirements. The storage demand grows exponentially with the number of errors considered, as a vast set of error patterns and syndromes must be stored. For example, correcting up to three errors in 1500-byte packets with CRC-24 would require approximately 2.6 TB of storage, while correcting four errors with CRC-32 would require nearly 10.4 PB [26], which is clearly impractical with current technology.

Statistical estimator approaches: Statistical estimators attempt to identify the most likely transmitted binary sequence, given the received erroneous sequence. A notable example is the maximum a posteriori (MAP) estimator, which can be combined with a CRC to evaluate the accuracy of the MAP-estimated sequence. These methods may leverage optimization techniques such as the alternating direction method of multipliers (ADMM) [29] or belief propagation (BP) [30]. Unlike lookup-based methods, they rely on soft information, assigning reliability scores to received bits. However, their applicability to current system architectures is limited due to the high computational burden [21].

Motivated by these limitations, we adapt a neural network with reject option to the context of Bluetooth Low Energy error correction. This method avoids the use of large memory-intensive lookup tables or computationally expensive statistical estimators, while providing a flexible and scalable alternative. Specifically, we propose a strategy that yields three possible outputs: 0, 1, or rejection (*R*). The rejected pattern (*R*) is used to identify the position of transmission errors, while a CRC is employed to detect erroneous packets. The reject option is activated only when the CRC detects an error. In most cases, our method successfully identifies and corrects bit positions with a high error probability. The system employs CRC-24 for error detection and an ELM neural network to address channel fading. Our results demonstrate that packets of varying sizes in BLE communication yield excellent performance, balancing efficiency and robustness under conditions of multipath fading and noise corruption. Furthermore, we demonstrate that applying the proposed method to images transmitted through error-prone channels substantially enhances the visual quality and can restore corrupted content.

## 2. Preliminaries

To provide context for our proposal, we briefly introduce the concepts of multipath fading, classification with reject option (enabling binary classification with three outputs), the cyclic redundancy check algorithm for error detection in data packets, and the extreme learning machine neural network.

### 2.1. Channel Fading and Equalization

Fading channels model the behavior of real-world signal propagation in wireless communication. These models account for multipath scattering, time dispersion, and Doppler shifts. At the receiver, non-resolvable components of the signal converge five rise to the phenomenon known as multipath fading. As a result, each significant propagation path can be modeled as a discrete fading path, leading to a complex channel response that varies over both time and frequency [31].

**Figure 2:**
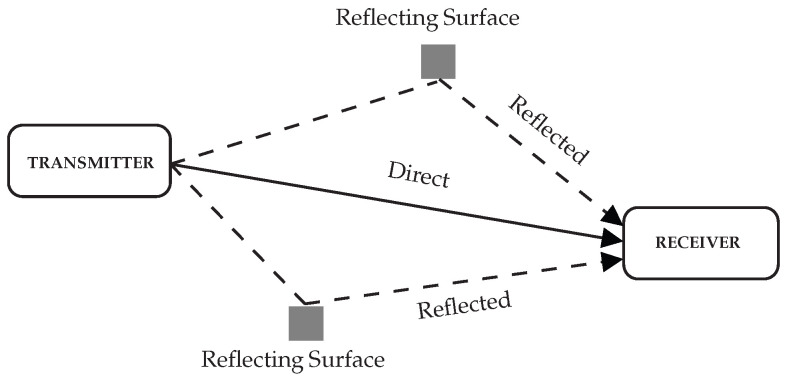
Multipath channel in wireless communications scenario.

Figure 2 illustrates the direct and reflected paths connecting a stationary radio transmitter to a stationary radio receiver. The shaded areas represent reflective surfaces, such as walls, which contribute to multipath effects by scattering signals. This scenario is prevalent in urban environments, where buildings and structures introduce significant path loss and shadowing effects.

### 2.2. Cyclic Redundancy Check

**Figure 3:**
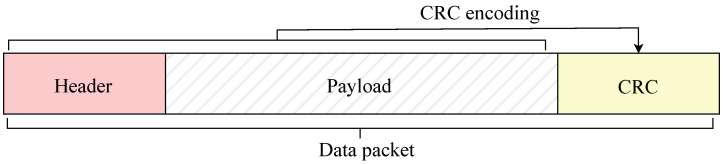
CRC-based framing.

In digital communication systems, data are typically divided into frames or packets for transmission, with each packet appended with a cyclic redundancy check code. CRC is a widely used and efficient method for error detection in data communication. It appends additional bits, known as check bits, to the end of the transmitted data [32]. At the receiver, the CRC value is recomputed and compared with the appended check bits. If the two values match, the packet is interpreted as error-free; otherwise, a transmission error is detected. Figure 3 illustrates an example of a packet framing scheme.

The CRC code is defined by a generator polynomial g(x), as shown in Table 1. The choice of generator polynomial is critical, as it determines both the length and the error-detection capabilities of the check bits. Different communication systems adopt different generator polynomials depending on their performance requirements. For example, the CRC-24-BLE code is the standard used in Bluetooth Low Energy communication.

### 2.3. Classification with Reject Option

Classification with a reject option encompasses methods designed to improve the reliability of decision support systems. These approaches were first formalized in the context of statistical pattern recognition, as described in [13], and are rooted in minimum risk theory.

Conventional classification methods assign every pattern to a class, even in uncertain situations, such as when a pattern lies close to the decision boundary. However, forcing classification under uncertainty may degrade the overall performance. A more prudent strategy is to classify only when sufficient confidence exists, rather than categorizing all patterns indiscriminately. Otherwise, accuracy control becomes compromised, and the classifier may exhibit suboptimal behavior, since its primary objective is usually to minimize the error rate [33].

Consider a binary classification problem, where N=2, and the classes are denoted as $\left\{C_{1},C_{2}\right\}$. In this setting, a reject option introduces a third class, $C_{reject}$, such that each sample is assigned to $C_{1}$, $C_{2}$, or $C_{reject}$. Patterns deemed too uncertain or complex to classify are placed into the reject class based on a decision threshold.

According to Chow [13,34], classifiers with reject options can be designed by minimizing the empirical risk:

(1) $$r_{\mathrm{emp}}=E_{rate}+\alpha R_{rate},$$

where $E_{rate}$ denotes the misclassification rate, $R_{rate}$ the rejection rate (both measured on validation data), and α the rejection cost, which must be predetermined by the user. A lower α encourages more rejections and reduces the error, while a higher α results in fewer rejections but an increased error rate.

For binary problems, classifiers with a reject option can be designed using three main approaches:

Method 1: A standard binary classifier is trained. If the maximum of the two posterior probabilities, $\max\{p\left(C_{1}|x\right),p\left(C_{2}|x\right)\}$, falls below a threshold *t* (0≤t≤1), the sample *x* is rejected. When the classifier does not provide probabilistic outputs, rejection is handled by applying a threshold to its output after training.

Method 2: Two independent classifiers are trained, one specialized in detecting $C_{1}$ when the probability of $C_{1}$ is sufficiently high and the other specialized in detecting $C_{2}$. The main advantage of this approach is its simplicity: a sample is rejected whenever the classifiers disagree.

Method 3: A single classifier is trained with a built-in reject option. In this case, the rejection region is incorporated during the training process itself. This approach requires learning algorithms capable of directly integrating a reject option into the optimization of their cost functions. The resulting model inherently learns to produce three possible outcomes: $C_{1}$, $C_{2}$, or $C_{reject}$.

### 2.4. Neural Networks

Neural networks are a class of machine learning models that take inspiration from the workings of the human brain to perform complex tasks efficiently. We find wide application in areas such as computer vision [35], speech recognition [36], natural language processing [37], and robotics [38].

Extreme learning machine features the ability to train a neural network with an input layer and a hidden layer without requiring the iterative backpropagation process, as it randomly initializes the weights in the hidden layer. As a result, the output layer weights are determined analytically without a parameter tuning process, using analytical methods to compute output weights [39].

Given the training set $D={\left\{\left(x_{i},y_{i}\right)\right\}}_{i=1}^{N}$ with *N* representing the number of patterns, $x_{i}={\left[x_{i1},x_{i2},\ldots ,x_{in}\right]}^{T}\in R^{n}$, and $y_{i}={\left[y_{i1},y_{i2},\ldots ,y_{im}\right]}^{T}\in R^{m}$, with *n* being the number of input features and *m* the number of categories, classes, or labels, *L* the number of hidden nodes, $w_{i}={\left[w_{i1},w_{i2},\ldots ,w_{in}\right]}^{T}$ is the weight vector connecting the *i*th hidden node and the input nodes, ${fi}_{i}={\left[\beta_{i1},\beta_{i2},\ldots ,\beta_{im}\right]}^{T}$ is the weight vector connecting the *i*th hidden node and the output nodes, $b_{i}$ is the threshold of the *i*th hidden node, and $g_{i}\left(.\right)$ is the activation function, $y_{j}$ is the output vector:

(2) $$y_{j}=\sum_{i=1}^{L}\beta_{i}g_{i}\left(x_{j}\right)=\sum_{i=1}^{L}\beta_{i}g_{i}\left(w_{i}\cdot x_{j}+b_{i}\right).$$

Here, j=1,…,N, and $w_{i}$ and $b_{i}$ are randomly defined weight vectors. H is called the hidden layer output matrix of the neural network. In matrix form it can be represented as follows:

(3) Hβ=Y,

where

$$H={\left[\begin{array}{cccc} g(w_{1}\cdot x_{1}+b_{1}) & \cdot \cdot \cdot & g(w_{L}\cdot x_{1}+b_{L}) & \\ \vdots & \cdot \cdot \cdot & \vdots \\ g(w_{1}\cdot x_{N}+b_{1}) & \cdot \cdot \cdot & g(w_{L}\cdot x_{N}+b_{L}) \end{array}\right]}_{N\times L},$$

$$\begin{array}{ccc} \beta ={\left[\begin{array}{c} \beta_{1}^{T}\\ \vdots \\ \beta_{L}^{T} \end{array}\right]}_{L\times m}, & & Y={\left[\begin{array}{c} y_{1}^{T}\\ \vdots \\ y_{N}^{T} \end{array}\right]}_{N\times m} \end{array}.$$

The output weight vector ${fi}_{i}={\left[\beta_{i1},\beta_{i2},\ldots ,\beta_{im}\right]}^{T}$ must be estimated in order to minimize the error function:

(4) $$\min{∥H\beta -Y∥}^{2}.$$

If the number of patterns *N* equals the number of neurons in the hidden layer *L*, N=L, the hidden layer output matrix H is square and invertible; therefore, ||Hβ−Y||=0 for any $w_{i}$ and $b_{i}$ randomly selected. However, in most training, the number of patterns is higher than the number of hidden layer neurons, N≫L, making H not square. Therefore, we present the least squares solution with the smallest norm as follows:

(5) $$\beta =H^{†}Y.$$

Here, $H^{†}$ can be calculated by a mathematical transformation with the Moore–Penrose generalized inverse of H represented as follows:

(6) $$H^{†}={\left(H^{T}H\right)}^{-1}H\;\mathrm{or}\;H^{†}=H^{T}{\left(HH^{T}\right)}^{-1}.$$

## 3. Proposed Method

**Figure 4:**
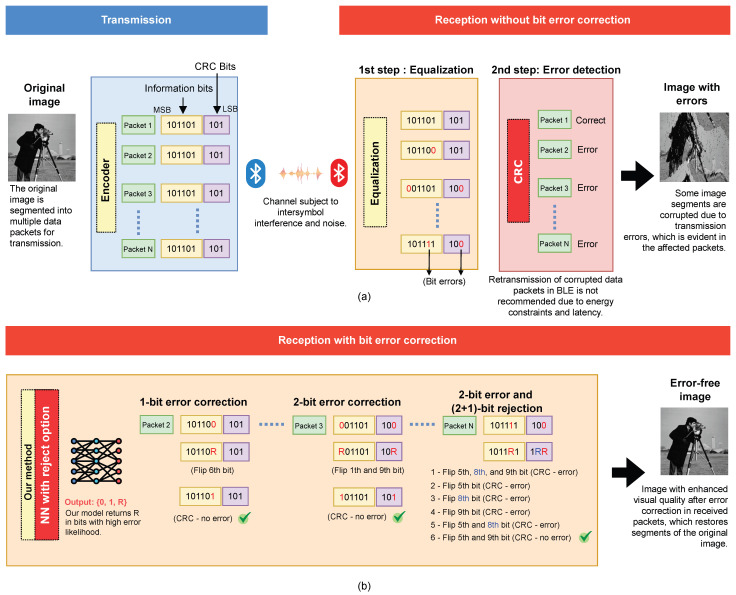
(**a**) An image transmitted via Bluetooth Low Energy is subjected to signal equalization upon reception, and the CRC validates the data packets. Packets 2, 3, and N are identified as containing errors. (**b**) Our method locates bit errors, flips the bit values at the positions marked as potential errors within the packets, and resubmits them for CRC verification, culminating in the successful reconstruction of the image.

When the cyclic redundancy check detects errors in a data packet, our correction method applies an extreme learning machine neural network with reject option, producing three possible outputs: 0, 1, and R. The R output is used exclusively for error identification and correction. When the NN detects patterns with a high error probability, it labels the bit as R. This marks the candidate error position, allowing our model to handle it explicitly. We then perform bit-value flips at positions labeled with R: if an R is assigned to a bit position, its value is inverted (0 becomes 1, and 1 becomes 0). After these modifications, we reapply the CRC to verify whether the packet still contains errors. Figure 4 illustrates detecting and correcting errors in a digital transmission using this procedure.

In our method, the rejection option is adapted to the BLE error correction scenario, directly linking Chow’s theoretical risk minimization to the practical identification of bit errors. Specifically, rejection is applied at the bit level whenever the neural network classifier exhibits low confidence, assigning the label R instead of forcing a binary decision {0,1}. Thus, the rejected bits represent candidate error positions, which are later explored during the CRC-guided correction process. This adaptation establishes a clear connection between the theoretical concept of empirical risk minimization and its practical use in recovering BLE packets under noisy and fading channels.

This section details the data modeling, training, prediction, and error correction processes of our neural network-based approach.

### 3.1. Data Modeling

A binary sequence of elements x∈{0,1} is used to train the neural network-based equalizer model. This sequence is represented as a vector $x=[x_{1},x_{2},\ldots ,x_{k}]$, where each $x_{i}$ denotes a modulated symbol, and *k* is the sequence length. The communication channel is characterized by the impulse response vector $h={\left[h_{1},h_{2},\ldots ,h_{L}\right]}^{T}$, where *L* is the number of channel taps.

The received signal can be initially described by the convolution of the transmitted sequence x with the finite impulse response (FIR) filter h. The convolution is expressed as

(7) v=h⊗x,

where ⊗ denotes linear convolution. In discrete form,

(8) $$v_{k}=\sum_{i=1}^{L}h_{i}x_{k-i}+\eta_{k},$$

where $\eta_{k}∼N\left(0,\sigma^{2}\right)$ represents additive white Gaussian noise (AWGN), and $x_{i}=0$ is assumed for i≤0.

In the case of Bluetooth Low Energy, the transmitted signal is Gaussian Frequency-Shift Keying (GFSK) modulated. It can be expressed in continuous time as

(9) $$s\left(t\right)=\exp\;\left\{j\left[2\pi f_{c}t+\pi h\;\;\int_{-\infty }^{t}\frac{1}{T}m_{G}\left(\tau \right)\;d\tau \right]\right\},$$

where $f_{c}$ is the carrier frequency, *T* is the symbol duration, h=0.5 is the modulation index adopted in BLE, and $m_{G}\left(t\right)$ denotes the binary sequence filtered by a Gaussian pulse-shaping filter. The impulse response h represents multipath propagation in the time domain, while its Fourier transform H(f) reveals that the channel is frequency-selective over the 1 MHz bandwidth of a BLE channel.

Finally, considering both multipath fading and external interference in the 2.4 GHz ISM band, the effective received signal is better represented as

(10) $$r_{k}=\sum_{i=1}^{L}h_{i}s_{k-i}+I_{k}+\eta_{k},$$

where $I_{k}$ accounts for interference from coexisting wireless technologies (e.g., Wi-Fi, ZigBee, or other BLE devices), and $\eta_{k}$ denotes AWGN. This extended representation highlights the practical challenges of BLE transmission and justifies the adoption of a neural network-based equalizer, designed to generalize effectively under frequency selectivity, interference, and noise.

### 3.2. Training a Neural Network with Reject Option

The process of building a classifier with a reject option involves several steps: (i) the relevant dataset, in this case the bits, is collected and split into a training and a testing set; (ii) the binary classifier is trained by adjusting the weights of the neural network connections. In this step, one can choose any neural network with reject option support, and (iii) the rejection threshold is trained, which is a crucial aspect of developing the classifier with three outputs as zero, one, or rejection, utilizing the concepts of the optimal decision rule. The process evaluates the binary classification model and rejection thresholds on the test set, using previously unseen data. From this evaluation, we derive optimal classification rules by balancing the accuracy and rejection rates. The optimal model and rejection threshold are selected, and integrating these findings helps to establish the connection weights, hyperparameters, and the rejection threshold, thereby automating the binary classifier’s third output.

We choose a neural network with reject option based on a single classifier structure, where a pattern is rejected if the maximum of the two binary estimates is less than a rejection threshold. The process involves fitting the connections by feeding the input data $x_{i}$, where i=1,2,3,…,N, and *N* is the number of patterns, into the model. The approach follows a strategy of finding the best threshold for a given cost function α, where α is the cost associated with the method. The cost function is evaluated for α, an input parameter in $\left[0.04,0.48\right]$. The decision threshold *t* impacts the number of rejected samples, where *t* is an input parameter in $\left[0,0.5\right]$.

In this approach, the prediction function is used for each threshold value to determine the class labels of the test patterns and check the number of classified and rejected classes. The functions compute the error and rejection rates, which are applied to the optimal decision rule. The most appropriate way to find the rejection rate is by using the following formulas:

(11) $$R_{rate}=\frac{\mathrm{total}\;\mathrm{number}\;\mathrm{of}\;\mathrm{rejected}\;\mathrm{patterns}}{\mathrm{total}\;\mathrm{number}\;\mathrm{of}\;\mathrm{patterns}},$$

and the error rate is

(12) $$E_{rate}=\frac{\mathrm{total}\;\mathrm{number}\;\mathrm{of}\;\mathrm{misclassified}\;\mathrm{patterns}}{\mathrm{total}\;\mathrm{number}\;\mathrm{of}\;\mathrm{patterns}};$$

this process is repeated for different values of α and *t* and chooses the one that minimizes the empirical risk:

(13) $$r_{emp}\left(\alpha ,t\right)=E_{rate}\left(t\right)+\alpha R_{rate}\left(t\right).$$

### 3.3. Prediction in a Neural Network with Reject Option

The prediction function assigns a class label to a given input pattern. The possible outputs are class 0 ($C_{1}$), class 1 ($C_{2}$), or rejection (*R*).

For a binary classification problem, the network produces two outputs, $y=[y_{1},y_{2}]$. The predicted class is determined by selecting the index of the maximum value. Specifically, if $y_{1}>y_{2}$, the pattern is classified as class 0 ($C_{1}$); otherwise, it is classified as class 1 ($C_{2}$):

(14) $$C_{y}=\left\{\begin{array}{cc} 0 & \;\mathrm{if}\;y_{1}>y_{2},\\ 1 & \;\mathrm{otherwise}.\; \end{array}\right.$$

After determining the predicted class $C_{y}$, the acceptance–rejection mechanism is applied based on a decision threshold *t*. The final output *C* is defined as

(15) $$C=\left\{\begin{array}{cc} C_{y} & \;\mathrm{if}\;\max(y)\geq 1-t,\\ R & \;\mathrm{otherwise}.\; \end{array}\right.$$

Thus, the prediction *C* can take three possible values:

I. C=0, if the pattern is most likely to belong to class $C_{1}$;
II. C=1, if the pattern is most likely to belong to class $C_{2}$;
III. $C=R(C_{reject})$, if neither class receives a sufficiently confident estimate to justify classification.

### 3.4. Error Detection and Correction Process

Cyclic redundancy check plays a central role in maintaining the flow of data packets by identifying packets that contain errors. If the CRC detects no errors, the data flow proceeds normally. However, when an erroneous packet is identified, our approach intervenes by classifying the bit sequence, marking positions where rejections (R) occur, and correcting the suspected bits. It is important to note that rejecting a pattern (bit) does not necessarily indicate that it is incorrect within the data packet. In a bit sequence, multiple errors and rejections may occur, requiring a combinatorial process to determine the positions at which the bit errors should be corrected.

**Figure 5:**
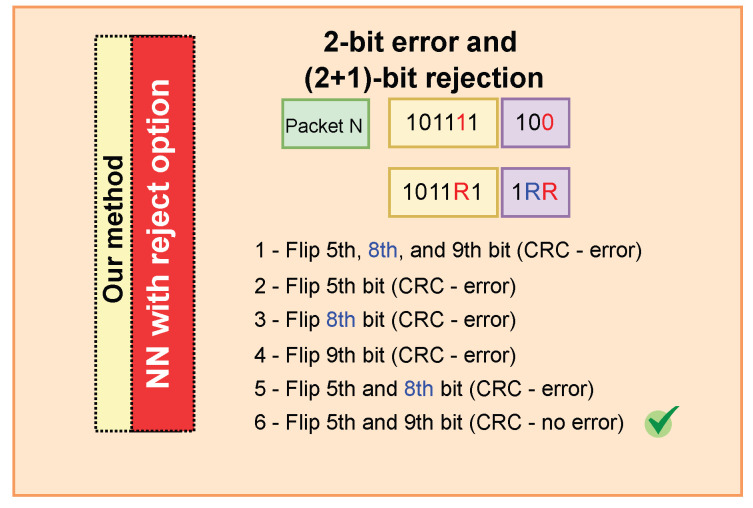
Demonstration of error correction in a BLE data packet with two-bit errors, using a combinatorial approach to invert bits at rejected positions.

Figure 5 presents a case of correcting a packet with two-bit errors. The CRC error detector indicates an error in this data packet. Our method suggests potential rejections in the fifth, eighth, and ninth positions, although only two bits are actually incorrect. A combinatorial procedure is then applied to identify the pair of positions that resolves the packet. In the first combination, the fifth, eighth, and ninth bits are inverted (i.e., 0 is changed to 1 and vice versa), but the CRC still reports an error; in the second combination, only the fifth bit is inverted, and the CRC still reports an error; in the third and fourth combinations, only the eighth bit and then the ninth bit are inverted, respectively, and in both cases the CRC indicates an error; in the fifth combination, the fifth and eighth bits are inverted, and an error remains; in the sixth attempt, only the fifth and ninth bits are inverted, and the CRC reports no error. In this case, the packet is corrected by the proposed method.

Note that the proposed method can handle errors of up to *n* rejected bits in a data packet. However, a high value of *n* leads to more candidate correction combinations and a higher computational cost to complete the correction. The additional complexity of our method arises from two components: (i) the neural network inference with a reject option and (ii) the combinatorial procedure that enumerates corrections over the rejected bits. Algorithm 1 is designed to correct packets by enumerating combinations of potential errors, where comb(i,j) denotes the matrix that contains all possible combinations of elements from vector *i* taken *j* at a time.
**Algorithm 1** Correcting bit errors in data packets with our approach.**Input:** Packet bit sequence seq, rejected positions rejected_pos**Output:** Correct error positions corrected_errors  1:corrected_errors ←∅  2:max_num_errors ←countRin rejected_pos where (size of rejected_pos ≤ n)  3:**for** num_errors ← 1 **to** max_num_errors **do**  4:$\;\;\;\;\;\;\begin{array}{c} comb \end{array}\leftarrow$ all combinations of R of size $\begin{array}{c} num\_errors \end{array}$  5:      **for** i←1  **to** $\mathrm{size}\;\mathrm{of}\;\begin{array}{c} comb \end{array}$ **do**  6:            seq_corrected ← seq  7:            **for** j←1 **to** num_errors **do**  8:                  Flip $\begin{array}{c} comb \end{array}\left[i\right]\left[j\right]$ in seq_corrected  9:            **end for**10:            **if** CRCof seq_corrected equals 0 **then**11:                  corrected_errors $\leftarrow \begin{array}{c} comb \end{array}\left[i\right]$12:                  **break**13:            **end if**14:       **end for**15:**end for**16:**if** corrected_errors ≠∅  **then**17:      “Error(s) found at positions”: corrected_errors18:**else**19:      “Unable to find the errors.”20:**end if**

## 4. Experiments and Results

We carried out simulations of data packet transmission over an error-prone channel to assess the gains achieved by our method relative to established statistical estimator approaches for error correction. Specifically, we benchmarked our approach against the CRC-ADMM and CRC-BP algorithms, with the goal of demonstrating its performance advantages. Our method uses an extreme learning machine neural network with reject option, which supports the classification of received signals and the correction of errors by detecting and handling rejected patterns.

Bluetooth Low Energy is widely used in Internet of Things (IoT) applications for efficient sensor data transmission [40]. In our simulations, we modeled end-to-end BLE channel transmission, explicitly accounting for multipath fading effects [41].

We consider a conventional receiver structure typical of BLE systems. The received signal is impaired by multipath fading and AWGN, which introduce intersymbol interference. The main challenges associated with this receiver model are the rapid variations of the wireless channel, which increase the bit error rate. Nevertheless, the extreme learning machine neural network with reject option operates as an equalization stage, identifying bits with a high probability of error.

Details regarding the neural network hyperparameters and the reject option configuration are provided in Table 2. The extreme learning machine was configured with a hidden layer ranging from 20 to 200 neurons, as this interval provides a balance between accuracy and computational efficiency in channel equalization tasks. The sigmoid activation function was chosen due to its ability to model nonlinear distortions introduced by multipath fading and noise. For training, we used sequences of $10^{5}$ bits per signal-to-noise ratio (SNR) value, while maintaining efficiency during training. Each experiment was repeated 20 times with a five-fold cross-validation scheme to reduce variance and validate generalization capability. Regarding the reject option, the rejection cost (α) varied from 0.04 to 0.48, and the decision threshold (*t*) from 0.00 to 0.50, allowing us to explore a wide spectrum of operating points between the rejection rate and the error rate. A higher cost parameter results in fewer rejections but increases the risk of misclassification, whereas a lower cost yields more rejections, which is advantageous under high-noise conditions. In our neural network with reject option, we adopt Chow’s optimal decision rule, which establishes the trade-off between the rejection rate and accuracy. Finally, we considered maximum rejection values (*n*) of 6, 8, and 10, corresponding to different error-correction capacities. These configurations were selected to provide a comprehensive analysis of the trade-off between the correction capability and computational complexity.

To further evaluate the robustness and adaptability, we conducted a series of experiments assessing the quality of image reconstruction under different conditions. All simulations were executed on a desktop computer equipped with an Intel Xeon CPU (2.3 GHz) and 16 GB of RAM.

### 4.1. Error Correction in Data Packets

This simulation evaluated the bit error rate and packet error rate (PER) for different modes of Bluetooth Low Energy packet transmission in the physical layer (PHY). Our experiments involved multiple configurations, including radio-frequency (RF) impairments, additive white Gaussian noise (AWGN), and Rayleigh fading. During the simulation, we varied the channel conditions by adjusting the signal-to-noise ratio, the data size in bytes, and enabling equalization to reproduce different BLE transmission scenarios.

For analysis, we used SNR values ranging from 0 dB to 18 dB, corresponding to BERs from $10^{0}$ to $10^{-5}$. The maximum size of a BLE packet is 261 bytes. However, we considered the effective payload size of the BLE packet, accounting for specific header information for each transmission, including headers, payloads, and cyclic redundancy check. We experimented with packets of 16, 32, 64, 128, and 256 bytes. We configured two samples per symbol and used an uncoded PHY with a data rate of 1 Mbps (LE1M) for the transmission. This setup allowed us to simulate the behavior of BLE transmission and obtain information about the system’s performance under different channel conditions and packet configurations.

**Figure 6:**
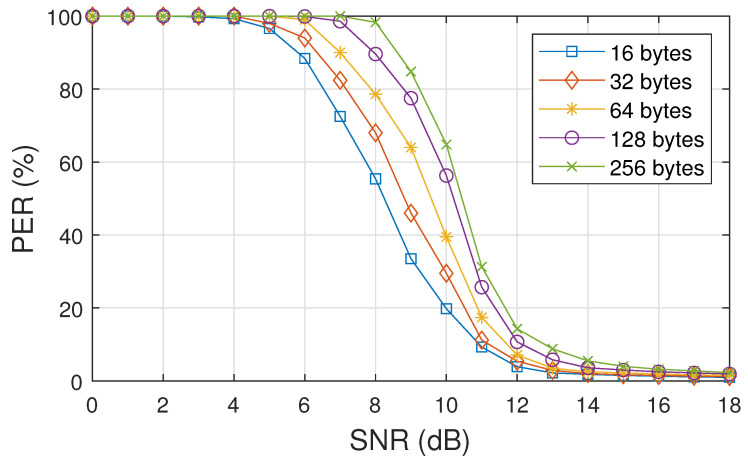
PER versus SNR for BLE LE1M transmission with packet sizes of 16, 32, 64, 128, and 256 bytes under AWGN and Rayleigh fading conditions.

In Figure 6, the PER versus SNR curve is presented for the simulated model, considering packets with different sizes. This curve allows us to assess how the packet error rate varies with SNR for different packet sizes. This information is important for understanding how packet size can impact transmission performance and communication quality.

We observed that, during packet transmission, longer packets exhibited a higher probability of error, whereas shorter packets were more resilient to lower SNR values. Considering Figure 6, each SNR (in dB) corresponds to a BER, defined as the ratio between the number of erroneous bits and the total number of transmitted bits. This BER reflects the probability of a single bit being received incorrectly under the given channel conditions. The packet error rate is then obtained by evaluating the impact of these bit errors on an entire packet of size *N* bytes. If the BER is low (few bit errors), the probability of a packet containing at least one error increases with the packet length, since more bits imply more opportunities for at least one error to occur. The packet error rate is therefore related to the bit error rate by the following equation:

(16) $$\mathrm{PER}\approx 1-{\left(1-\mathrm{BER}\right)}^{8N},$$

where *N* denotes the packet size in bytes (overhead + payload).

**Figure 7:**
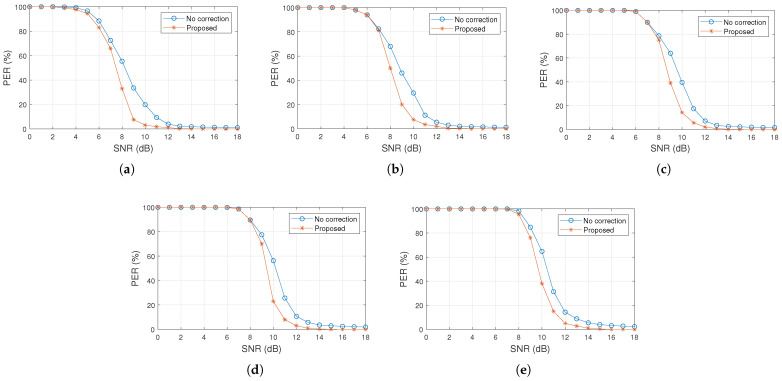
PER performance under two scenarios: packets with bit errors and the proposed method capable of correcting a subset of erroneous packets. Results are shown for different packet sizes: (**a**) 16 bytes, (**b**) 32 bytes, (**c**) 64 bytes, (**d**) 128 bytes, and (**e**) 256 bytes.

Figure 7 presents the simulation results for packet correction, considering the PER versus SNR curves. The figure shows the curve corresponding to packets without error correction methods, along with the results obtained using the proposed method, which is capable of correcting a subset of erroneously detected packets. Overall, the proposed method proved effective from an SNR of 4 dB, converging more rapidly toward a near-zero PER compared to transmission without packet error correction. Furthermore, the results indicate that smaller packet sizes yield greater efficiency for the proposed model in the tested scenarios.

In Figure 7a, for an SNR of 10 dB and a packet size of 16 bytes, the PER was 20%, while with the proposed model it was reduced to 4%. In Figure 7d, with an SNR of 10 dB and a packet size of 128 bytes, the PER is 56%, whereas the proposed method reduces it to 23%. In Figure 7e, with an SNR of 10 dB and a packet size of 256 bytes, the PER is 64%, whereas the proposed method reduces it to 38%. Similarly, in simulations with an SNR of 10 dB and packet sizes of 32 and 64 bytes, the PER values are 30% and 40%, respectively, while with the proposed method they decrease to 8% and 15%, respectively.

To further evaluate the practical relevance of the proposed method, we analyzed the effective throughput under the BLE PHY rate of 1 Mbps using 128-byte packets at 10 dB SNR. Taking into account the protocol overhead (preamble, access address, header, and CRC), the net payload efficiency was approximately 92.7%, resulting in a maximum achievable throughput of 0.927 Mbps. Without correction, the high packet error rate (PER = 56%) reduces the effective throughput to about 0.41 Mbps. In contrast, when the proposed error correction is applied (PER = 23%), the throughput increases to approximately 0.71 Mbps. This gain demonstrates that the proposed approach not only improves reliability but also reduces the need for retransmissions, thereby enhancing the efficiency of BLE communications.

Considering that the proposed method employs a rejection parameter of n=6, this enables the correction of up to six bits per data packet. However, it is important to note that the relationship between errors and rejections is not necessarily one-to-one, meaning that the number of errors and the number of rejections may vary. Nevertheless, the rejected bits generate a list of potential error candidates. Algorithm 1 performs the correction of packets based on combinations of these possible errors.

Based on the evaluation conducted by [42], a relationship was observed between packet size and packet loss probability. This relationship arises from the fact that larger packets are more likely to be lost compared to smaller ones. In this study, 10,000 data packets were analyzed for each packet size, ranging from 16 to 256 bytes.

By considering different packet sizes and error levels, the performance of the proposed method could be comprehensively evaluated across multiple scenarios. Table 3 presents the bit error correction rate achieved by the implemented approach for different packet sizes and error counts, as obtained through simulation.

The results demonstrated that, for single-bit-error packets of 16, 32, 64, 128, and 256 bytes, the correction rates reached 98.1%, 96.9%, 95.8%, 94.1%, and 93.6%, respectively, with only a modest reduction as packet size increased. For packets with two-bit errors, the method maintained robust performance, with correction rates ranging from 68.7% to 54.3%. Even in the presence of three-bit errors, the proposed approach successfully recovered a considerable portion of packets, achieving correction rates between 29.7% and 25.1%. Although the performance decreases for packets with four or more errors, the method still recovers a subset of packets, demonstrating resilience beyond the single-error regime. Furthermore, increasing the number of rejections, denoted as *n*, can further enhance the correction rates, although at the cost of additional computational resources due to the extra processing required.

### 4.2. Image Bit Error Correction

**Figure 8:**
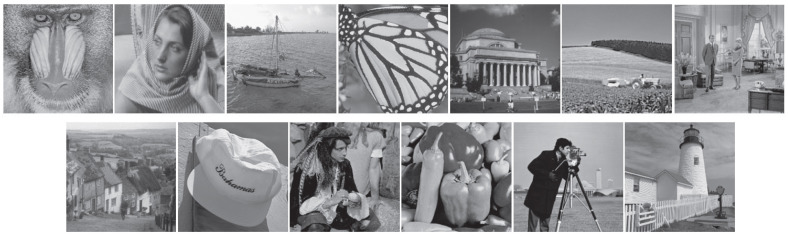
The 13 test images employed in our experiments are arranged in a grid from left to right and top to bottom: Baboon, Barbara, Boat, Butterfly, Columbia, Cornfield, Couple, Goldhill, Hat, Man, Peppers, Cameraman, and Tower [19].

We used 13 images that are widely recognized as benchmark test sets. We chose to work exclusively with grayscale images because color images comprise multiple channels, which can be reconstructed by recovering those channels independently. All images used in the experiment have dimensions of 250×250 pixels, as shown in Figure 8. These images capture a variety of objects and scenes to ensure diversity and content complexity [19].

Images are represented as sequences of bits organized into data packets. These packets were subjected to Huffman entropy coding to reduce redundancy and, consequently, decrease the image size without any loss of information. After compression, the packets are transmitted over a wireless communication system. However, when traversing a noisy channel, packets may be distorted by inter-symbol interference (ISI) and noise, resulting in reception errors. When such corrupted packets are decompressed, the embedded errors compromise image reconstruction, producing artifacts and a loss of visual quality. With our error-correction approach, the affected packets are identified and processed prior to decompression. The corrected packets are then decompressed, enabling image reconstruction with superior visual quality, in some cases approaching that of the original image.

We employed the Structural Similarity Index (SSIM) as an evaluation metric to assess image quality and quantify the gains provided by error correction. For each simulation, we computed the SSIM for both the received image without error correction and the image reconstructed using packet-level error-correction schemes. The SSIM is widely adopted for assessing image quality, as it measures the structural similarity between a reference image and a received (or reconstructed) image. Comparing SSIM values enables us to quantify the impact of error correction on our results, demonstrating how our approach improves the quality of reconstructed images relative to the absence of error correction:

(17) $$\mathrm{SSIM}\left(x,y\right)=\frac{\left(2\mu_{x}\mu_{y}+C_{1}\right)\;\left(2\sigma_{xy}+C_{2}\right)}{\left(\mu_{x}^{2}+\mu_{y}^{2}+C_{1}\right)\;\left(\sigma_{x}^{2}+\sigma_{y}^{2}+C_{2}\right)}.$$

In these expressions, *x* and *y* denote the two images being compared; $\mu_{x}$ and $\mu_{y}$ are the mean pixel intensities of *x* and *y*; $\sigma_{x}$ and $\sigma_{y}$ are the standard deviations of the pixel intensities; and $\sigma_{xy}$ is the covariance between the pixel intensities of *x* and *y*. The constants $C_{1}$ and $C_{2}$ are small positive stabilization terms to avoid division by zero. The SSIM value lies within [−1,1], where 1 indicates a perfect match between images, and −1 indicates complete dissimilarity. Values closer to 1 correspond to higher quality relative to the reference image.

We also employed the Peak Signal-to-Noise Ratio (PSNR) as a complementary metric to assess the effectiveness of our error correction approach. The PSNR is widely used in image quality evaluation, providing an objective measure of reconstruction accuracy by comparing the pixel intensity differences between the original and the processed image. Unlike the SSIM, which focuses on structural similarity, the PSNR quantifies the magnitude of the error in terms of signal strength relative to noise. The PSNR is expressed in decibels (dB) and is calculated as follows:

(18) $$\mathrm{PSNR}=10\cdot \log_{10}\left(\frac{{\mathrm{MAX}}^{2}}{\mathrm{MSE}}\right),$$

where MAX denotes the maximum possible pixel intensity value in the image (e.g., 255 for an 8-bit image), and the MSE (Mean Squared Error) is given by

(19) $$\mathrm{MSE}=\frac{1}{MN}\sum_{i=1}^{M}\sum_{j=1}^{N}{\left(x_{ij}-y_{ij}\right)}^{2},$$

where $x_{ij}$ and $y_{ij}$ represent the pixel intensities at position (i,j) in the original and processed images, respectively, and *M* and *N* denote the dimensions of the image. A higher PSNR value indicates lower distortion, meaning that the reconstructed image is closer to the original. By comparing both the SSIM and PSNR, we obtain a more comprehensive analysis of image quality. While the SSIM captures structural degradation, the PSNR provides insights into the absolute error levels, enabling us to assess the trade-off between perceptual quality and numerical accuracy in our image error correction approach.

Based on the error distribution reported in [41], we conducted experiments in noisy environments with an SNR ranging between 9 dB and 11 dB. In this configuration, most of the observed errors in the received packets were relatively minor, with 93% of the errors affecting fewer than three bits per data packet and 80% of the packets containing two or fewer errors, as presented in Table 4. This scenario provides an ideal setting for evaluating our error correction approach and its impact on visual quality.

Table 4 shows that the proportion of corrupted packets with a single error decreases as the SNR is reduced. This behavior is expected because, under noisier channel conditions, the likelihood of multiple bit flips within the same packet increases, redistributing the error statistics toward two or more errors. In contrast, at higher SNR values, corrupted packets are mostly limited to isolated single-bit errors.

We compared our approach with the CRC-ADMM and CRC-BP methods by evaluating the SSIM improvement rate on the tested images. These methods were selected because they do not rely on lookup tables and perform correction solely using the redundancy provided by the CRC. For the comparison, we examined packets of different sizes, including payloads of 8 bytes, 21 bytes, and 39 bytes, as used in the simulation conducted in [24]. This selection reflects common data transmission scenarios in IoT systems, where efficient error correction is critical for reliable communication.

Table 5 presents a comparison of the reconstructed images obtained using various error correction methods in a BLE environment, applied under different noisy conditions based on the SNR. In this table, the average considers all the tested images, taking into account the packet size, noise level, and the algorithm used. The results indicate that as the channel quality deteriorates, the images suffer from greater corruption. However, the proposed approach demonstrates significant improvements in image quality as assessed by the SSIM. For example, for 8-byte packets and the “Baboon” image with an SNR of 11 dB, our approach increased the SSIM value from 0.1261 to 0.9990, resulting in a visual gain of 0.8179 (81%), almost fully restoring the image. With an SNR of 10 dB, we observed an improvement of approximately 75%. Even under severe noise conditions with an SNR of 9 dB, where the image is heavily corrupted, our method still improves the visual quality by about 41% for the “Baboon” image.

It is worth noting that, in this simulation, we adopted the number of rejections equal to 6, 8, and 10, corresponding to SNR levels of 11 dB, 10 dB, and 9 dB, respectively. The choice of these values was aimed at evaluating the impact of the number of rejections under different noise conditions, especially in scenarios where the computational cost associated with additional processing can be offset by gains in error correction. A direct outcome of this evaluation is the marked decline in performance observed for the competing methods when the SNR is reduced to 10 dB and, in particular, to 9 dB.

When comparing the results of our proposed method with CRC-ADMM and CRC-BP, we observed that our approach achieved higher SSIM improvement rates in the experiments conducted. Furthermore, as the noise level increased, our correction method was more effective than the others, particularly in the simulation with an SNR of 9 dB. Since these gains vary across different images, packet sizes, and unfavorable channel conditions, the average SSIM results for the competing methods are detailed in Table 5.

**Figure 9:**
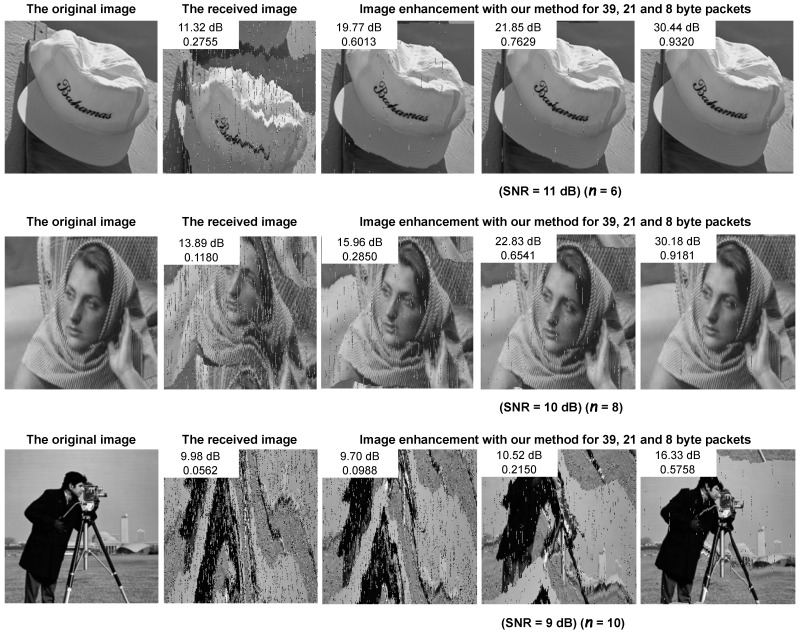
Comparison of image quality among the original image, the received image, and the image processed with our method after error correction, assessed across various packet sizes (39, 21, and 8 bytes) and SNR levels (11, 10, and 9 dB).

Figure 9 provides a visual example highlighting the benefits of our approach in terms of the packet size and robustness to noisy environments, considering BLE channels with SNRs of 9, 10, and 11 dB. Each example includes both the corrupted image and the reconstructed image. The corrupted image exhibits several distortions caused by errors, which significantly compromise its visual quality. In contrast, our approach demonstrated a remarkable ability to restore details and enhance the visual appearance of the received image. However, it is important to note that not all corrupted packets can be fully corrected, especially those of 21 and 39 bytes under an SNR of 9 dB, leading to more limited improvements. This limitation arises from the restricted size of BLE packets, as the probability of the occurrence of more than three errors increases under adverse channel conditions. Visually, the error-corrected images exhibited fewer artifacts and more natural structural details, which corroborates the improvements indicated by SSIM and PSNR.

Furthermore, we evaluated the computational efficiency of the proposed method in terms of the processing time for image correction, using the “Cameraman” image as an example under maximum correction capacity. The CRC-ADMM and CRC-BP methods require 252 s and 562 s, respectively, to correct a single image. In contrast, our approach achieved significantly faster results: 0.7 s for a maximum rejection count of n=6, 4 s for n=8, and 36 s for n=10. This total duration encompasses the combined time for error identification via the neural network with reject option and subsequent CRC-based verification within the packet. It is important to note that the correction mechanism is only activated when the CRC detects an error; if no error is present, no additional delay is introduced.

## 5. Discussion

The proposed approach introduces an error correction strategy that combines the cyclic redundancy check mechanism with an extreme learning machine with reject option. From the perspective of computational requirements, the integration of the reject option into the ELM does not significantly increase the inference costs. Once the rejection threshold is optimized during training, the prediction operates with comparable latency to a conventional classifier, ensuring feasibility for real-time communication systems. This contrasts with iterative methods such as ADMM and BP, whose decoding processes demand multiple iterations and are associated with higher execution times. In our experiments, packet correction was achieved in the order of microseconds, confirming that the method can operate under stringent latency constraints typical of BLE and IoT scenarios.

In terms of memory consumption, the method remains lightweight. Unlike LDPC and Turbo codes, which require storing large sparse matrices to achieve good performance, the proposed model relies only on the parameters of the ELM and the scalar rejection threshold. Additionally, the CRC mechanism requires minimal storage, limited to the checksum value computed for each packet. This makes the proposal highly attractive for devices with constrained memory and energy budgets, such as IoT sensors, where conventional error correction codes would be impractical.

Regarding overhead, the proposed scheme does not introduce redundant bits beyond those already included by the CRC in BLE communication. This contrasts with channel coding strategies such as Turbo, LDPC, and Polar codes, which add redundancy and reduce the effective code rate. Preserving bandwidth is particularly beneficial in BLE, where retransmissions increase latency and energy consumption. In the proposed method, the correction process occurs only when the CRC detects errors, which means that error-free packets incur no additional overhead. When errors are detected, the rejection mechanism guides the bit-flipping process with a limited number of candidate corrections, keeping additional computation bounded.

Concerning the error correction rate, simulations demonstrated high efficiency for single-bit errors, with correction rates above 94% across different packet sizes, and robust performance for two-bit errors, where correction ranged from 54% to 69%. Even in the presence of three errors, the system recovered up to 30% of corrupted packets, extending correction beyond the single-error regime typically guaranteed by lightweight coding strategies. These results highlight the method’s balance between reliability and low complexity. The proposed method contributes to the low-energy operation of BLE systems, since reducing the need for packet retransmissions directly lowers the energy expenditure at the transmitter, thereby extending battery lifetime. Furthermore, the improvements observed in image reconstruction experiments confirm the practical benefits of the approach in multimedia transmission, showing substantial gains in SSIM and PSNR, especially under adverse SNR conditions.

Although we limited our image evaluation to 13 benchmark test images, the scalability of the proposed model is not restricted to this dataset. The method operates at the packet level and does not depend on image-specific characteristics, meaning that any binary data stream (including larger image sets, video, or IoT sensor data) can be processed in the same manner. Regarding multi-bit errors, scalability can be achieved by adjusting the number of rejections (*n*), which allows correction of larger error patterns at the cost of increased computational complexity.

Internet of Things (IoT) devices typically rely on microcontrollers with limited processing power, which makes it challenging to execute large or complex neural networks in real time. This limitation motivated the adoption of the ELM model in our hardware experiments, as it provides fast inference with low computational overhead, making it particularly suitable for embedded applications. Beyond microcontrollers, however, more powerful platforms can extend the applicability of our method. For example, Field-Programmable Gate Arrays (FPGAs) enable parallelization and hardware-level acceleration of neural network inference, allowing real-time operation under higher throughput demands. These hardware options demonstrate that our algorithm can be efficiently implemented in resource-constrained IoT devices as well as in high-performance wireless communication infrastructures, underscoring its versatility for real-world deployment.

The proposed methodology is best suited for terrestrial short-range BLE communications, which are representative of IoT scenarios affected by multipath fading. At this stage, however, our focus remains on BLE systems, where the proposed framework demonstrates practical benefits in terms of reliability and efficiency. Nevertheless, the method can also be applied to other challenging wireless environments in future studies.

Overall, the proposed method effectively combines low computational and memory cost, absence of redundant-bit overhead, and high correction capability. This positions it as a competitive alternative to existing error correction strategies, particularly in low-power wireless communication environments where efficiency and real-time performance are critical.

## 6. Conclusions

This paper presented an application of a neural network with reject option for error correction in Bluetooth Low Energy systems. The proposed approach employs pattern rejection to effectively recover corrupted packets, underscoring the practical relevance of neural network-based systems with a reject option in data communication applications. We evaluated the method in the context of wireless data transmission using Bluetooth Low Energy technology. The experimental results demonstrated its ability to correct single-bit errors and double-bit errors in packets of varying lengths without relying on conventional error-correcting codes or introducing redundancy beyond the cyclic redundancy check mechanism. By reducing retransmissions and mitigating packet loss, the framework contributed to improved energy efficiency on the transmitter side. It achieved correction rates of 94–98% for single-bit errors, 54–68% for double-bit errors, and 26–29% for triple-bit errors. In image processing scenarios, the method also proved competitive, in many cases restoring the visual integrity of corrupted images and significantly enhancing the perceptual quality. Future research will aim to extend the framework toward autonomous error detection and correction, potentially reducing or even eliminating the need for conventional error-detection algorithms such as CRC. In addition, we plan to evaluate battery lifetime metrics with and without the proposed method in real BLE hardware.

## Figures and Tables

**Table 1:** Common generator polynomials.

CRC	Value
CRC-4-ITU (4 bits)	$g\left(x\right)=x^{4}+x+1$
CRC-8-CCITT (8 bits)	$g\left(x\right)=x^{8}+x^{2}+x+1$
CRC-16-CCITT (16 bits)	$g\left(x\right)=x^{16}+x^{12}+x^{5}+1$
CRC-24-BLE (24 bits)	$g\left(x\right)=x^{24}+x^{10}+x^{9}+x^{6}+x^{4}+x^{3}+x+1$

**Table 2:** Techniques and parameters for reproducing the approach and results.

Technique	Description	Values
ELM	Number of neurons per hidden layer Activation function Number of patterns Executions Cross validation using the k-fold	20:20:200 Sigmoid $10^{5}$ bits per dB 20 5
Reject option	Rejection cost (α) Decision threshold (t) Maximum number of rejections (n)	0.04:0.04:0.48 0.00:0.01:0.50 6, 8, 10

**Table 3:** Error correction rate for different packet sizes and number of errors (%).

	Packet Size (Bytes)				
Errors	16	32	64	128	256
1 error	98.1	96.9	95.8	94.1	93.6
2 errors	68.7	64.9	61.7	55.1	54.3
3 errors	29.7	28.5	27.2	25.8	25.1
>3 errors	1.9	1.3	0.9	0.2	0.2

**Table 4:** Average number of errors per corrupted packet for different channel conditions (%).

	SNR Value		
Errors	11 dB	10 dB	9 dB
1 error	85.1	76.5	53.3
2 errors	12.2	13.5	27.4
3 errors	2.1	4.8	13.0
>3 errors	0.6	5.2	6.3

**Table 5:** Average SSIM comparison among various images and packet sizes (PS) (8, 21, and 39 bytes) under different BLE channel conditions. The following correction methods were tested: ➀: Image Reception (with Errors), ➁: CRC-BP, ➂: CRC-ADMM, and ➃: Proposed Method.

Images	PS	SNR = 11 dB (*n* = 6)				SNR = 10 dB (*n* = 8)				SNR = 9 dB (*n* = 10)			
		➀	➁	➂	➃	➀	➁	➂	➃	➀	➁	➂	➃
Baboon	8 Bytes	0.1261	0.3102	0.8882	**0.9990**	0.0832	0.3538	0.5962	**0.8401**	0.0614	0.0601	0.0656	**0.4762**
	21 Bytes	0.1507	0.5326	0.2742	**0.8220**	0.0797	0.1870	0.2562	**0.2805**	0.0653	0.0648	0.0604	**0.1184**
	39 Bytes	0.1352	0.1470	0.5465	**0.4663**	0.0856	0.1138	0.1388	**0.1650**	0.0725	0.0617	0.0760	**0.0650**
Barbara	8 Bytes	0.2084	0.8190	0.7520	**0.9999**	0.1383	0.6199	0.7092	**0.9181**	0.0714	0.0765	0.0729	**0.4809**
	21 Bytes	0.2193	0.6801	0.4486	**0.7187**	0.1254	0.3436	0.3132	**0.6541**	0.0650	0.0698	0.0767	**0.2236**
	39 Bytes	0.2342	0.4137	0.4380	**0.4585**	0.1263	0.2304	0.2075	**0.2850**	0.0773	0.0748	0.0766	**0.1081**
Boat	8 Bytes	0.1964	0.3981	0.3880	**0.8500**	0.0780	0.2663	0.3634	**0.5719**	0.0421	0.0542	0.0384	**0.4725**
	21 Bytes	0.1925	0.3721	0.3241	**0.6590**	0.0718	0.3268	0.1600	**0.3288**	0.0495	0.0522	0.0483	**0.1506**
	39 Bytes	0.1317	0.2326	0.3860	**0.4725**	0.0861	0.1615	0.1506	**0.1486**	0.0421	0.0448	0.0492	**0.0718**
Butterfly	8 Bytes	0.0862	0.4202	0.7263	**0.9936**	0.0560	0.2749	0.3315	**0.3964**	0.0326	0.0275	0.0233	**0.4419**
	21 Bytes	0.1104	0.5374	0.2425	**0.6248**	0.0412	0.1378	0.2483	**0.1144**	0.0326	0.0232	0.0272	**0.0681**
	39 Bytes	0.0871	0.1265	0.1136	**0.4715**	0.0482	0.1082	0.0874	**0.1039**	0.0181	0.0218	0.0312	**0.0373**
Columbia	8 Bytes	0.2317	0.5526	0.5367	**0.8234**	0.1377	0.6539	0.7468	**0.9250**	0.0748	0.0852	0.0668	**0.4919**
	21 Bytes	0.2762	0.5798	0.6307	**0.6459**	0.1524	0.3216	0.5130	**0.5023**	0.0788	0.0758	0.0807	**0.2893**
	39 Bytes	0.2623	0.4521	0.5452	**0.6507**	0.1628	0.2694	0.2114	**0.2575**	0.0716	0.0839	0.0933	**0.0953**
Cornfield	8 Bytes	0.2565	0.5752	0.6089	**0.8798**	0.1641	0.5057	0.5941	**0.6282**	0.0830	0.0839	0.0757	**0.4674**
	21 Bytes	0.2326	0.6290	0.5660	**0.5081**	0.1459	0.3947	0.3902	**0.3813**	0.0796	0.0786	0.0803	**0.2411**
	39 Bytes	0.2868	0.4050	0.4005	**0.5636**	0.1507	0.2357	0.2467	**0.3043**	0.0727	0.0874	0.0873	**0.1016**
Couple	8 Bytes	0.2661	0.6622	0.5181	**0.8359**	0.1559	0.6995	0.5834	**0.4792**	0.0886	0.1002	0.0751	**0.4261**
	21 Bytes	0.2924	0.4469	0.4152	**0.5649**	0.1796	0.4617	0.3780	**0.4520**	0.0761	0.0837	0.0826	**0.2641**
	39 Bytes	0.3097	0.3608	0.4628	**0.4155**	0.1484	0.2729	0.2603	**0.3494**	0.0855	0.0819	0.0818	**0.1165**
Goldhill	8 Bytes	0.1770	0.3577	0.7885	**0.9975**	0.1320	0.4677	0.8129	**0.7406**	0.0688	0.0645	0.0632	**0.4471**
	21 Bytes	0.2087	0.4692	0.4653	**0.6051**	0.1161	0.2384	0.2247	**0.4066**	0.0516	0.0644	0.0519	**0.1199**
	39 Bytes	0.2113	0.5291	0.4699	**0.4838**	0.1003	0.2627	0.1787	**0.2603**	0.0556	0.0723	0.0707	**0.0905**
Hat	8 Bytes	0.3534	0.5973	0.7636	**0.9320**	0.1669	0.6700	0.6065	**0.5173**	0.0853	0.0817	0.0909	**0.4524**
	21 Bytes	0.2739	0.5454	0.6903	**0.7629**	0.1543	0.4427	0.4034	**0.5824**	0.0871	0.0892	0.1033	**0.2449**
	39 Bytes	0.2649	0.4663	0.5504	**0.6013**	0.1660	0.2473	0.2616	**0.3455**	0.0883	0.0778	0.0904	**0.1279**
Man	8 Bytes	0.1000	0.3324	0.6541	**0.5659**	0.0475	0.5917	0.3545	**0.5219**	0.0275	0.0304	0.0459	**0.2790**
	21 Bytes	0.0768	0.4078	0.5683	**0.6442**	0.0498	0.3352	0.1799	**0.1657**	0.0394	0.0346	0.0316	**0.0783**
	39 Bytes	0.1091	0.1971	0.3029	**0.2199**	0.0505	0.0908	0.0633	**0.1875**	0.0417	0.0438	0.0385	**0.0505**
Peppers	8 Bytes	0.2850	0.5240	0.6449	**0.9992**	0.1354	0.7675	0.6455	**0.6607**	0.0420	0.0391	0.0426	**0.4860**
	21 Bytes	0.2786	0.5673	0.6397	**0.6319**	0.1394	0.4656	0.3313	**0.3946**	0.0540	0.0386	0.0391	**0.1070**
	39 Bytes	0.2586	0.4172	0.4331	**0.4764**	0.0851	0.3198	0.1803	**0.3434**	0.0364	0.0440	0.0352	**0.0585**
Cameraman	8 Bytes	0.2867	0.6703	0.7991	**0.7793**	0.1434	0.6366	0.5447	**0.6796**	0.0592	0.0677	0.0680	**0.5758**
	21 Bytes	0.2915	0.6985	0.5789	**0.6327**	0.1565	0.4042	0.3984	**0.5724**	0.0641	0.0605	0.0676	**0.2150**
	39 Bytes	0.3024	0.5407	0.5041	**0.4910**	0.1246	0.2387	0.2744	**0.3620**	0.0605	0.0567	0.0542	**0.0988**
Tower	8 Bytes	0.2554	0.4919	0.7859	**0.7422**	0.1331	0.5699	0.8024	**0.7233**	0.0617	0.0741	0.0672	**0.5150**
	21 Bytes	0.2752	0.7309	0.4984	**0.6642**	0.1651	0.4337	0.4135	**0.5124**	0.0602	0.0617	0.0717	**0.2083**
	39 Bytes	0.2496	0.4767	0.4930	**0.5897**	0.1273	0.2484	0.2739	**0.3358**	0.0665	0.0672	0.0653	**0.1035**
**Average**	8 Bytes	0.2176	0.5162	0.6811	**0.8767**	0.1208	0.5445	0.5916	**0.6617**	0.0615	0.0650	0.0613	**0.4624**
	21 Bytes	0.2214	0.5536	0.4879	**0.6526**	0.1213	0.3456	0.3239	**0.4113**	0.0618	0.0613	0.0632	**0.1792**
	39 Bytes	0.2187	0.3665	0.4343	**0.4892**	0.1125	0.2154	0.1949	**0.2652**	0.0607	0.0629	0.0654	**0.0865**

## Data Availability

The original contributions presented in this study are included in the article. Further inquiries can be directed to the corresponding author.
